# Supplementary material for: Are carbon emissions trading and green financial instruments synergistic? -Comprehensive quantitative research based on content analysis
Source: PLoS One. 2024 Mar 7;19(3):e0298601. doi: 10.1371/journal.pone.0298601 (PMC10919601; doi:10.1371/journal.pone.0298601)
Supplement: S1 Data — (ZIP) [file pone.0298601.s001.zip › Data/地级市Z_F工作报告.zip!/░▓╗╒/┴∙░▓╩╨/└┤╫╘╣1⁄2╓┌║┼í╛┬φ┐╦╩2╛▌═°í┐_2015.docx]

各位代表：
　　现在，我代表市人民政府，向大会报告工作，请予审议，并请市政协委员和其他列席人员提出意见。
　　一、幸福六安建设迈出坚实步伐
　　2014年，全市人民在市委的坚强领导下，认真贯彻党的十八大和十八届三中、四中全会精神，坚持稳中求进、改革创新，全力提质增效、转型升级，统筹做好稳增长、促改革、调结构、惠民生的各项工作，攻坚克难，砥砺奋进，保持了经济社会稳定健康发展。预计实现生产总值1091亿元，增长7.5%；财政收入142.1亿元，增长11.3%；城镇常住居民人均可支配收入20378元，增长9.2%；农村常住居民人均可支配收入8354元，增长11%。
　　一年来，主要做了以下八个方面的工作：
　　**第一，强化政策落实，稳定经济增长。**全面贯彻落实中央及省稳增长促改革调结构惠民生的各项政策措施，支持实体经济和小微企业发展，安排财政专项资金3.88亿元，减免缓抵各项税费17.2亿元，新增规模以上工业企业61户、总数达1010户；预计实现规模以上工业增加值440亿元，增长10%。稳定发展粮食生产，实现粮食总产92.6亿斤，连续11年丰收；我市首获“全国粮食生产先进市”称号，霍邱、寿县再获“全国粮食生产先进县”称号。加快推进农产品加工“168”工程，预计实现加工产值820亿元，增长10%。积极培育新型农业经营主体，新增农民专业合作社1624家、家庭农场970户，其中国家级农民专业合作社示范社13家、省级8家、省级示范家庭农场12户。积极扩大信贷投放，金融机构各项贷款余额958.2亿元，增长16.6%，其中新增贷款135亿元。实现直接融资21亿元，应流机电成功上市，迎驾贡酒在审待发，龙华竹业股改即将完成，恒瑞新能源在“新三板”挂牌，14家企业在区域性股权交易市场挂牌，15家企业签订“新三板”辅导协议。新设立7家村镇银行，中信银行六安分行即将开业。
　　**第二，协调需求拉动，增强发展后劲。**按照“经济工作项目化、项目工作责任化”要求，推动项目工作提质提效，预计完成固定资产投资1000亿元，增长18.5%；其中，市“253”工程亿元以上项目新开工180个、续建252个，完成投资610亿元，增长15.7%。进一步提高招商引资质量和水平，预计实际到位境内资金755亿元、增长15%，直接利用外资3.4亿美元、增长15%。六安电厂二期、金寨信义光伏、华润怡宝水一期等项目建成投产，新加坡联发创意生态工业园、中财管道、中创信测科技产业园、中至信家具等项目开工建设，中兴金寨战略性新兴产业园、大别山旅游综合服务中心等项目相继落户。大力促进消费增长，预计实现社会消费品零售总额488亿元，增长12.5%。继续加快“新网工程”、“五位一体便民服务工程”等城乡流通体系建设，新建、改造城乡农贸市场12个。大力发展电子商务，六安电子商务创业园建成运营，霍山县被列为全国电子商务进农村综合示范县。扎实推进快递物流园区建设，皖江吉宝冷链物流园、六安中财智能公路港物流等项目开工建设。落实稳定外贸增长政策，开展系列经贸活动，预计实现进出口总额7.36亿美元。
　　**第三，致力绿色发展，加快转型升级。**实施绿色发展战略，编制《六安市生态文明建设规划》，启动“六安茶谷”建设，成功举办“绿色发展生态六安论坛”、“绿色发展跨洲视频连线国际研讨会”；5县区被列入国家首批生态文明先行示范区—巢湖生态文明先行示范区，霍山、金寨2县被列入国家主体功能区建设试点示范。实施创新驱动发展战略，新认定国家高新技术企业34家、省级高新技术产品 103个，新增省级企业技术中心6家、省级创新型企业5家；全年申请发明专利1060件，增长45%；授权发明专利100件。启动六安大学科技园建设，引进项目40个。新增国家地理标志保护产品1个、中国驰名商标2个、安徽名牌产品17个。大力培育战略性新兴产业，预计实现产值255.9亿元，增长15.7%。加快开发园区转型升级，霍邱经济开发区被列为全国循环化改造示范试点园区。整顿矿业开发秩序，整改企业56家、注销67家。积极推广绿色建筑，完成30万平方米试点建设。促进旅游业持续发展，成功举办“2014年全国群众登山健身大会暨第三届中国大别山（六安）山水文化旅游节”、“第十届中国安徽雪花啤酒节”等活动，全年接待游客2412万人次，实现旅游总收入163亿元，分别增长20%、25%，霍山县成功创建全国休闲农业与乡村旅游示范县。加强资源节约和环境保护，大力推进扬尘治理、秸秆禁烧等大气污染防治工作，城区烟花爆竹限放工作初显成效。继续实施森林增长工程，新增造林面积26.97万亩。
　　**第四，全面深化改革，激发市场活力。**启动市县政府机构改革，市级政府机构改革方案获批；完成县级市场监管体制改革任务，市级食品药品监管体制改革职能交接顺利完成。继续深化行政审批制度改革，及时衔接落实国务院和省政府取消、下放的行政审批事项，市级行政审批事项减少至136项。推进商事制度改革，进一步降低工商注册准入门槛，市场活力和社会创造力有效激发。加强土地节约集约利用，顺利通过国家土地出让金专项审计和省土地节约集约利用督察，成功列入全国第二批工矿废弃地复垦利用试点市。基本完成金寨、金安2县区和其他县区7个乡镇农村土地确权登记颁证试点工作；开展农村集体产权制度改革试点，积极搭建流转交易平台，推动土地承包经营权、集体建设用地使用权、农村房屋产权、林权等有效流转。农村合作金融机构改革基本完成，金寨、六安、叶集农商行开业运营，其他县区农商行全部获批；金寨县农村金融综合改革通过全国人大中期评估，取得阶段性成果。启动政策性农业保险市场化改革和特色农业保险工作。实施新一轮市级国有企业改革，完成48家企业清产核资和11家企业脱钩改制。深化行政事业单位国有资产管理改革，启动市直生产经营服务类事业单位转企改制。积极探索政府购买服务，实施流浪乞讨人员救助、社区建设等14个项目试点。推进财税体制改革，调整完善市与区财政管理体制，加强政府性债务管理和涉农资金监管。做好“营改增”扩围试点，累计兑现财政扶持资金4315.6万元。
　　**第五，统筹城乡发展，促进区域协调。**成功创建省文明城市，国家森林城市建设总体规划通过国家林业局专家评审。完成六安东部新城暨核心区总体规划、西部生态新区规划以及淠河总干渠生态治理和景观规划，开展城市南部发展战略研究。进一步加快中心城市建设，共实施项目83个、完成政府性投资49.6亿元，凤凰桥污水处理厂、苏大堰河道治理工程、清水河畔棚改安置小区等37个项目竣工。强力推进征迁工作，中心城区重点工程征迁面积219.2万平方米。加大土地经营力度，房地产市场平稳运行。启动中心城区工业企业“退城进园”工作。开展“三治、三增、三提升”活动，加快推进县城和中心集镇建设，完成霍邱县、金寨县新一轮县城总体规划修编，舒城、霍邱、寿县建设中等城市步伐加快，霍山“智慧城市”试点扎实推进。大力推进美好乡村建设，首批83个重点示范村通过省考核验收，启动第二批87个省级中心村建设。大力开展“三线三边一点一地”城乡环境整治，城乡面貌明显改善。加快交通基础设施建设，完成投资70亿元，新建和续建国省干线公路项目25个，G312六安段（二、三期）、六舒路（一期）、大别山旅游扶贫快速通道金寨段（一期）主体工程建成通车；济祁高速寿县段加快建设；新建和升级县乡公路345公里，改造危桥66座；舒城县在全市率先实现城乡公交一体化。深入推进农村电网和通讯设施改造升级，农网改造线路2203公里。加强耕地保护，新增耕地2万余亩，完成高标准基本农田建设79万亩；治理水土流失面积110平方公里，除险加固小Ⅱ型水库110座。深入推进皖江示范区建设，全面加强合肥经济圈合作，寿县蜀山现代产业园建设步伐加快，舒城包河现代产业园筹建工作有序推进。大力推进精准扶贫，减少贫困人口14万人。
　　**第六，坚持民生优先，发展社会事业。**认真实施省、市37项民生工程，投入财政资金73.5亿元。加大就业工作力度，城镇新增就业10.18万人，城镇登记失业率控制在3.96%以下。城乡社会保障制度体系不断完善，保障水平逐步提升。7类“老字号”群体补助发放工作进展顺利，农村危房改造和“渔民上岸”集中安置点建设加快推进。开工建设各类保障性住房和棚户区改造安置房31189套，基本建成13974套，新增发放租赁补贴945户。加快公共文化服务体系建设，市图书档案馆开工建设，新增群众文化广场示范点40个，建成农民文化乐园15个，公共文化服务场所免费开放。霍山县被评为全国文化先进县，“八公山豆腐”传统制作工艺入选第四批国家级非物质文化遗产目录，3项文艺作品获全省“五个一”工程奖。成功承办中韩文化交流活动。启动实施城区义务教育阶段公办学校建设三年行动计划，5所新建、改扩建小学加快建设或投入使用；一中东校区、二中西校区建设加快推进，毛坦厂中学东部新城校区建设启动。六安职业技术学院各县区分院组建挂牌，金寨职业学校建成招生。成功承办中韩青少年体育交流活动，全民健身运动蓬勃开展，六安代表团在省十三运会上取得历史最好成绩。基层医改成果进一步巩固，医疗卫生服务能力显著提高，新批准民营医疗机构4家。出生人口性别比稳步下降，人口自然增长率7.4‰。顺利完成第三次全国经济普查工作，统计基础工作进一步加强。国防动员训练基地开工建设，民兵预备役和双拥工作扎实推进。人防、档案、地方志、民族宗教、外事侨务、气象、妇女儿童、老龄、残疾人等各项事业取得新进展。
　　**第七，创新社会治理，促进社会和谐。**坚持领导干部开门接访、带案下访、包案处访，开展“无越级上访乡镇（街道）” 创建活动，及时解决群众反映强烈的问题。社区（村）网格化服务管理实现全覆盖。建立重点领域行业性专业性调解组织160多个，调解成功率达90%。积极开展“六五”普法，加强法律援助和社区矫正安置帮教工作，霍山县荣获“全国青少年普法教育示范区”。圆满完成村“两委”换届工作。依法管理互联网，确保网络和信息安全。健全应急管理体制机制，做好防灾减灾工作，应对突发事件的预防预警和处置能力得到有效提升。扎实推进安全生产体系建设，安全生产形势总体平稳，基本完成烟花爆竹生产企业整体退出任务。深入开展食品药品安全专项整治，保障人民群众食品药品安全。着力推进农村平安服务网等十项重点工程建设，加快建设立体化、数字化社会治安防控体系，严厉打击各类违法犯罪活动，人民群众安全感、满意度不断提升。
　　**第八，加强政府建设，严格依法行政。**深入开展党的群众路线教育实践活动，严格践行“三严三实”，政府系统“四风”问题得到有力整治，群众反映强烈的突出问题得到切实解决。严格执行中央八项规定和国务院“约法三章”，市级“三公”经费支出下降21.1%。自觉接受人大监督，依法执行人大决议决定，认真办理安置房建设与管理、政策性农业保险等人大代表议案和建议156件。主动接受政协民主监督，积极吸纳“加快绿色发展”等调研成果，办理政协提案248件。密切与工会、共青团、妇联等人民团体的联系，高度重视舆论监督和社会公众监督。进一步规范行政决策行为，大力推进政务公开。规范执法行为，畅通行政复议和行政调解渠道。推进公共权力运行监控平台建设，首批5家市直单位29项行政审批权纳入平台监控。建立涉企收费清单制度，市级收费项目减少32.9%。开展规范性文件清理和非行政许可审批清理工作，进一步规范企业投资项目前置审批条件。加强和改进政府投资项目、财政预算执行、领导干部经济责任及其他专项资金审计，加大稳增长等政策措施落实情况跟踪审计力度，强化问题整改。全面推进惩治和预防腐败体系建设，强化行政监察，廉政建设取得新成效。
　　各位代表，过去的一年，面对加快发展的艰巨任务和经济下行的巨大压力，我们咬定目标，坚定信心，克难进取，合力攻坚，有效应对各种困难和挑战，保持了经济社会持续健康发展，成绩来之不易。这是省委、省政府和市委科学决策、正确领导的结果，是市人大、市政协和社会各界全力支持、有效监督的结果，是全市上下同心同德、奋力拼搏的结果。在此，我谨代表市人民政府，向全市各界人士表示诚挚的感谢!
　　在肯定成绩的同时，我们也清醒看到，我市经济社会发展还存在不少矛盾和问题。主要是：经济下行压力加大，部分经济指标增幅回落，未完成年度目标任务；部分骨干企业效益下滑，城乡居民增收乏力；科技创新能力不强，现代服务业发展滞后；投资、消费、出口有效需求减弱，发展后劲有待增强；土地、用工、资金等要素制约依然突出，节能减排和环境保护任务艰巨；政府自身建设还需进一步加强，“四风”问题还没有从根本上得到解决，违纪违法问题还时有发生。对此，我们将高度重视，采取有效措施，切实加以解决，不辜负全市人民和各位代表的期望和重托。
　　二、奋力冲刺“十二五”目标任务
　　2015年是全面深化改革的关键之年，也是奋力冲刺“十二五”目标任务的收官之年，做好今年工作意义重大。当前，世界经济仍处在国际金融危机后的深度调整期，我国经济发展进入速度变化、结构优化、动力转换的新常态。为此，我们要深刻认识新常态、主动适应新常态、积极引领新常态，变压力为动力，顺势而为，乘势而上，奋力开创全市经济社会发展新局面。
　　今年政府工作的总体要求是：全面贯彻落实党的十八大和十八届三中、四中全会精神，主动适应经济发展新常态，坚持以幸福六安为总目标、以绿色发展为总取向、以稳中求进为总基调，把转方式调结构放到更加重要位置，着力推动改革攻坚，着力推进自主创新，着力扩大有效投入，着力加强生态建设，着力保障改善民生，着力建设法治政府，努力保持经济持续健康发展和社会和谐稳定。
　　综合考虑，2015年主要预期目标是：全市生产总值增长8%左右，财政收入增长10%左右，规模工业增加值增长11%左右，固定资产投资增长15%左右，社会消费品零售总额增长13%左右，城镇、农村常住居民人均可支配收入分别增长9%和10%左右，城镇登记失业率控制在4.5%以内，人口自然增长率控制在7.6‰以内，“十二五”节能减排任务全面完成。
　　实现上述目标，必须着重抓好以下八个方面的工作：
　　（一）全力促进经济平稳增长
　　**推动项目建设提质增效。**持续扩大有效投入，充分发挥投资对经济发展的关键作用。组织编制“十三五”规划，统筹推进项目谋划储备、计划编制、调度考核等工作，着力提高重大项目谋划成功率、开工率、竣工率、达产率。力促首矿大昌300万吨铁矿深加工、佛子岭抽水蓄能电站等项目复工建设，加快中财管道、胜利精密制造、吉宝冷链物流、叶集家居产业园、悠然蓝溪、亚夏汽车财富广场、合六南通道、济祁高速六安段、商合杭高铁寿县段、淠河史河治理等项目建设，积极推进应流高端装备制造、云母深加工应用、金寨抽水蓄能电站、迎驾产业园、霍山石斛产业园、合六叶高速拓宽改造、金寨钼矿等项目尽快开工，大力推进六安电厂三期、合安九客运专线、六安景铁路等项目前期工作。
　　**提高招商引资质量和水平。**围绕产业发展方向和主导产业定位，积极承接技术含量高的新兴产业和市场前景好的劳动密集型产业，加大现代服务业招商引资力度，力争实现到位境内资金和外商直接投资分别增长10%。大力开展产业链招商，推动上下游产业集聚发展，引进培育一批区域性总部、功能性机构和研发中心。深化与央企、知名民企合作，强化与商会、协会对接。守住土地和环保红线，严把投资强度关和环境评价关。强化跟踪服务，健全推进机制，完善考核办法，提高签约项目履约率、资金到位率和开工率。
　　**大力促进消费和进出口。**完善消费政策，实施养老健康家政、信息、旅游、住房、教育文化体育等领域消费工程，加快培育新的消费增长点。积极培育商贸龙头企业，大力发展限上企业，有序推进万达广场、浙东商贸城等城市综合体项目建设或运营。深入实施“便民服务体系建设工程”，着力打造15分钟便民消费圈；完善农村商品流通服务体系。大力发展电子商务，加快淘宝“特色中国•六安馆”建设，推动六安快递物流园区、大别山电子商务产业园建设。落实外贸促进政策，加大对进出口企业支持力度，扎实开展外贸企业包保服务，力争进出口总额增长10%。精心举办第五届中国（六安）国际羽绒博览会，积极组织企业参加各类商务会展，利用国际市场开拓、信保补贴等政策资金，推动企业扩大出口。
　　**强化金融支撑。**创新金融产品和服务，扩大信贷投放规模，引导金融机构重点投向主导产业、新兴产业和重大基础设施等领域，加大对小微企业支持力度，切实提升金融服务“三农”和实体经济发展水平。着力加强担保体系建设，做强各级国有背景的担保公司，完善银政担合作机制，探索建立专项担保、风险补偿及过桥接续专项资金。大力推进直接融资，强化后备资源培育，争取全年新增上市企业1家、“新三板”挂牌企业3家以上、区域性股权交易市场挂牌企业10家以上。培育、支持设立股权投资基金和创业投资基金。加强信用体系建设，扩大信用评级结果运用，优化金融生态环境。
　　（二）加快绿色发展步伐
　　**强化规划引领。**认真实施《安徽省主体功能区规划》和《六安市生态文明建设规划》，进一步明确重点开发区域、限制开发区域和禁止开发区域，着力构建绿色发展布局体系。加快制定《六安市水土保持规划》，合理划定水土流失重点预防区、重点治理区，落实生态空间开发管制。以节能环保和资源循环利用为目标，加大对现有产业的生态化改造力度，构建新型绿色产业体系。加快编制“六安茶谷”发展规划，扎实推进项目实施，统筹茶、竹、石斛等特色农业及旅游文化产业发展。建立符合绿色发展要求的评价考核、监管和责任追究制度，加快构建绿色发展制度体系。
　　**加强生态建设和环境保护。**实施《六安市生态市建设规划》，推动巢湖生态文明先行示范区创建和霍山、金寨国家主体功能区建设试点示范工作，支持舒城县创建国家级生态县。实施生态修复和治理工作，切实加强自然保护区、风景名胜区、生态功能区和矿产资源开发区的生态保护、建设与修复，加快瓦埠湖等环境综合治理项目实施进度。加大集中式饮用水水源地保护力度，实行最严格水资源管理制度。制定科学合理的补偿方案，开展大别山区（淠河流域）水环境生态补偿工作。推进森林增长工程和绿色长廊建设。大力推进集约节约用地，扎实做好土地例行督察准备工作。落实能源消费强度和总量目标责任，严格实施项目能评制度。加快节能技改重点项目建设，推广节能产品，推动重点行业节能降耗。大力推广绿色建筑，新增建筑面积 60万平方米。深入开展工业废气、城市扬尘、机动车尾气、秸秆焚烧等大气污染防治专项行动，强化水污染、土壤污染防治。
　　**加快发展现代服务业。**实施服务业发展规划，加快六安市城市商业网点规划修编，全面完成县级城市商业规划编制工作；加快各县区服务业重点板块及集聚区建设，增强省级服务业集聚区辐射带动能力。大力发展现代物流、金融商务、邮政服务、科技咨询、总部经济等产业。积极发展与现代制造业相结合的云计算、大数据、物联网等新兴业态。大力发展文化产业，加快推进大别山玉石产业园、皋陶文化园等重点项目建设。加强规划管控，实施六安市旅游产业重点项目三年行动计划，加快六大旅游区、五条特色旅游线路、旅游集散中心体系、旅游信息平台建设，完善基础设施配套。支持万佛湖、佛子岭争创5A级景区。加强旅游产品开发和营销体系建设，推进旅游与文化、生态、健康等产业深度融合。设立和引进旅游产业投资基金，鼓励社会资本投资旅游业。加强旅游市场监管，推进旅游诚信体系建设。
　　**增强科技创新能力。**培育壮大战略性新兴产业，重点发展高端装备制造、电子信息、节能环保产业，积极培育生物、新材料、新能源产业，力争产值达到300亿元。着力提升企业自主创新能力，培育壮大一批科技领军型创新企业，引导龙头企业自建或联合科研院所、高等院校共建工程技术中心。加强政策引导，提高专利创造质量，力争全年发明专利申请量达1100件以上、授权量达115件以上。加快六安大学科技园建设，完善落实各项政策措施，制定入园孵化企业条件和标准。认真实施科技富民强县专项行动计划，不断完善农村科技服务体系，推进农村科技进步。
　　（三）全面深化改革
　　**深入推进行政体制改革。**进一步加大简政放权力度，衔接落实好国务院和省政府取消、下放的行政审批事项，继续削减压缩市级行政审批事项，清理和简化前置审批条件，提高审批效率。加强事中事后监管，做到放管结合、放而不乱。深化行政执法体制改革，重点在市场监管、公共资源交易、交通运输、城乡建设等领域内推行综合执法。全面建立和推行市县两级政府权力清单、责任清单和涉企收费清单制度，进一步激发市场主体活力。完成市县政府机构改革，加快事业单位分类改革，推进市直生产经营服务类事业单位转企改制。深化工商登记制度改革，做好企业信息公示工作。推进政府购买服务，扩大购买范围。
　　**深化农村综合改革。**稳妥有序推进农村土地制度改革，认真抓好金寨全国农村土地改革试点，深入开展土地承包经营权和农村房屋所有权确权登记颁证工作，完成舒城、霍山、裕安农村土地确权登记工作。严格规范农村土地流转行为，发展适度规模经营和现代种养业，完善服务体系，强化用途监管。积极推进农村集体产权制度改革，加快农村产权流转交易市场建设，提高集体经济组织资产运营管理水平。推进农民住房财产权抵押、担保、转让试点。深化小型水利工程产权制度改革。推进集体林权制度配套改革。加快政策性农业保险市场化改革，稳步推进特色农业保险工作。支持金寨县作为全国第二批农村改革试验区，积极开展农村合作金融组织试点。
　　**促进财税金融改革。**完善政府预算体系，建立跨年度预算平衡机制，进一步细化政府预决算和部门预决算公开内容；加强政府债务管理，防范债务风险；探索推广政府与社会资本合作(PPP)模式，积极推动融资平台转型发展；清理规范税收优惠政策，健全全社会协税护税机制；继续实施“营改增”改革。加快推进国资国企改革，实现国有资产效益最大化和效率最优化。加快普惠金融体系建设，支持农村商业银行规范发展、壮大实力，更好发挥地方法人金融机构生力军作用；支持民间资本通过多种形式进入金融业，大力引进域外金融机构，实现村镇银行县区全覆盖；深化金寨县农村金融综合改革，完成金安区农村金融改革试点任务，全面推开其他县区的改革工作。
　　**深化文化体制改革。**健全完善文化管理体制，推动政府部门由办文化向管文化转变。推动国有文化企业进行公司制、股份制改造，建立现代企业制度，支持转制国有文艺院团加快发展。鼓励文化企业跨地区、跨行业、跨所有制兼并重组，加快培育多层次的文化产品和要素市场，切实提高企业资本运作和市场经营能力。改革公共文化服务投资体制，鼓励社会力量和社会资本参与公共文化服务体系建设。健全坚持正确舆论导向的体制机制，加快推动传统媒体与新兴媒体融合发展。
　　**推进教育卫生体制改革。**深化教育领域改革，加快构建优质普惠性学前教育体系，统筹城乡义务教育资源均衡配置。探索中考中招改革，完善示范高中招生制度。规范民办中小学办学管理，鼓励社会力量兴办教育。推进医药卫生体制改革，深化城市公立医院综合改革，在市、县两级公立医院推行“药品零差率、分级诊疗”服务模式。推进城乡基层医疗卫生服务网络化，提升基层卫生计生服务能力。积极扶持中医药事业发展，鼓励社会办医。加快医保支付方式改革，完善药品招标配送机制，减轻群众就医负担。
　　（四）推进新型工业化
　　**做大工业经济总量。**全面落实中央、省促进工业经济发展的政策措施，加大市级支持力度，加强跟踪问效，确保贴息、贴费、退税、奖励等各项政策落到实处。强化工业运行监测调度，加强对重点行业、企业、产品的监测分析，及时研究解决苗头性、趋势性问题。加大企业帮扶力度，深入研究重点企业帮扶解困方案，有效解决融资、市场开拓等难题。加快培育骨干企业，鼓励有条件的企业通过扩建改造、联合重组、股改上市等方式做大做强，力争新增规模工业企业50户，工业投资增长20%。
　　**引导企业创新发展。**强化技术创新，提升企业技术改造、科技研发、工业设计能力，力争完成技改投资220亿元，支持应流集团建立国家级企业技术中心，引导15家企业建立省、市级企业技术中心。加大新产品研发力度，力争新认定10项以上省级新产品。加强质量工作，实施品牌战略，积极创建“全国知名品牌示范区”和“全省优秀产品示范区”。大力推行重点行业清洁生产，发展循环经济，提高资源综合利用率。加强企业信息化建设，新培育20户以上省、市两化融合示范企业。
　　**加快发展民营经济。**全面落实支持民营经济发展政策措施，放宽准入条件，降低企业创办门槛，鼓励有条件的个体工商户、家庭农场向民营企业转型发展。继续实施千名民营企业家培养计划，提升企业家管理水平和综合素质。搭建和完善中小企业服务平台，加强小微企业创业示范基地建设，推动民营企业健康发展。
　　**推动园区转型升级。**按照产城一体的要求，优化开发园区空间布局，完善园区基础设施和配套服务业。严格项目准入标准，严把产业规划、环境保护、投资强度和税收贡献关，完善园区考评体系。加强新型工业化产业示范基地建设，加快推进城区工业企业“退城进园”。推进多层厂房、标准化厂房建设，强化招商、运营。创新园区开发模式，引导社会资本开发经营工业地产、建设园中园。搭建园区信息服务平台，推广信息化技术示范应用，积极争创“智慧园区”、省级两化融合示范园区。加快实施霍邱经济开发区循环化改造项目。
　　（五）力推新型城镇化
　　**加强中心城市规划建设与管理。**健全完善城乡规划体系，推进城市控制性详细规划和各类专业规划编制进度，强化重点区域城市设计。组织编制绿色城南规划，启动交通路网、生态景观等基础设施建设。继续实施中心城市重点工程建设，计划政府投资38.8亿元，重点加快六安火车站及站前广场、赤壁路桥、图书档案馆、文化科技馆等87个项目建设。统筹推进东部产业新城、西部生态新区建设，着力完善配套、提升品位。继续加大征迁拆违工作力度，保障重点工程建设。巩固深化省级文明城市创建成果，深入开展国家森林城市、环保模范城市、节水型城市创建。建立城市管理标准化体系，加快数字化城管、信息基础设施和应用平台建设，提升城市管理精细化水平。推进城管综合执法，理顺执法体制。强化源头治理，深入开展超载超限、渣土运输等专项整治。加大土地经营力度，规范土地市场秩序，保障建设用地需求。推动房地产市场平稳健康发展，不断提高物业管理服务水平。
　　**大力提高县城和乡镇规划建设水平。**推动城镇规划编制和依法实施，加强规划衔接，推进“多规合一”试点。加快县城建设步伐，重点推进道路建设、污水和垃圾处理、园林绿化、水环境治理，不断改善人居环境，充分发挥县城吸纳农村转移人口的重要作用。深入开展县城管理提升行动，提高县城创建水平。统筹城乡发展，重点加强中心集镇建设，完善基础设施，打造一批规模合理、功能完善、产业配套、管理规范的特色集镇。
　　**抓好美好乡村建设。**深入推进第二批87个省级中心村建设，启动实施第三批76个中心村建设。突出乡村特色，抓好村庄规划编制、区域产业布局工作，着力打造一村一景、人文独特、别具魅力的美好乡村。积极发展农村集体经济，注重培育特色产业，促进农民增收致富。建立健全长效管护机制，确保实现长治久美。深入开展“三线三边一点一地”城乡环境综合整治，加大工作力度，着力拓展治理区域，提升治理水平。
　　**推进区域协调发展。**以全境享受皖江示范区政策为契机，加快承接产业转移步伐。深度融入合肥经济圈，加快推进交通基础设施、市场一体化、大气联防联治等合作专题，推动合六南通道全线开工建设，加快六安-合肥城际轨道交通前期工作，推动寿县蜀山现代产业园、舒城包河现代产业园加快建设，谋划启动示范园区、霍邱县与合肥市共建合作园区。深化皖北结对合作，扩大基础设施、产业发展、招商引资、社会管理等合作领域，继续推进寿县、霍邱供肥蔬菜基地建设，完善两县至合肥旅游精品路线建设，加快建设以寿县新桥国际产业园为载体的航空港经济示范区。
　　（六）加快农业现代化
　　**增强农产品综合供给能力。**全面落实国家粮食安全政策措施，创新粮食生产支持保护机制和风险防范机制。加快推进粮食生产“三大行动”和高产创建示范片建设，狠抓关键技术措施落实，稳步提高粮食产能。深入推进“粮安工程”和粮食仓储设施建设，促进粮食转化利用和减损增效。组织实施蔬菜产业提升行动和百万亩油茶产业发展行动计划，加快茶产业转型升级。完善畜禽水产良种繁育体系建设，加快发展现代渔业。培育壮大农业特色产业，有效发掘利用六安瓜片、皖西白鹅、霍寿黑猪、霍山石斛、水库有机鱼、大别山油茶、毛竹等品牌产业资源，提高规模种养比重、品质和效益。建立健全农产品质量安全监管体系，强化食用农产品产地管理，鼓励企业争创“三品一标”，完善质量安全承诺和追溯制度。
　　**构建新型农业经营体系。**加大农民合作社、家庭农场和专业大户培育发展力度，强化技术、人才、资本等要素对接。大力扶持农业生产社会化服务组织，推进农民合作社向联合社发展。鼓励家庭农场牵头或参与组建农民合作社，培育一批与城市社区、农产品交易市场、零售商场合作的家庭农场。深入开展示范合作社和示范家庭农场创建活动，实行动态监测管理，促进科学经营和规范发展。整合涉农项目资金，优先扶持新型农业经营主体发展。
　　**大力推进农业产业化。**做大做强农产品加工业，力争产值达到1000亿元。新增亿元以上龙头企业15家，重点培育100个左右龙头企业。着力引进市外、省外农业产业化龙头企业。鼓励龙头企业自建优质农产品原料生产基地，推广应用农业物联网技术。支持农产品加工企业技术研发和升级改造，提升精深加工水平。加强龙头企业风险监测和预警处置，开展银企对接和金融征信服务，支持企业多渠道融资。
　　**夯实农业发展基础。**加大“三农”政策支持和投入保障力度，组织实施重点农业项目。加强农村实用人才队伍建设，培养新型职业农民。推进农业科技创新工程，健全完善公益性和社会化相衔接的服务机制。强化现代农业产业技术体系建设，积极争创国家级现代农业园区和农产品加工示范区。加强农村基础设施建设，继续实施小型农田水利改造提升行动计划。加强耕地保护，科学划定永久性基本农田，统筹推进土地整治项目，新增高标准基本农田75.2万亩。
　　（七）保障和改善民生
　　**巩固提升民生工程。**坚持保基本、兜底线、促公平，进一步调整财政支出结构，加大财政资金向民生倾斜力度，认真实施好省、市37项民生工程。完善考核评价办法，加强绩效管理，提高民生工程建后管养水平。加快保障性安居工程建设，开工建设各类保障性住房和棚户区改造安置房31306套，基本建成18630套。扎实推进老旧小区综合改造，持续改善居民生活环境。改革城市公交、出租车运营体制，提升运行能力和服务质量，更好方便市民出行。
　　**完善就业服务和社会保障体系。**积极开展大众创业，推动以创业带动就业，支持促进代工产业发展；深入实施就业促进工程和农民工职业技能提升计划，切实抓好就业困难家庭、高校毕业生、军队随军未就业家属、复退军人等群体就业工作，推进农村富余劳动力就地就近转移就业。推进社会保险扩面征缴，重点推进农民工、灵活就业人员等群体参保工作。继续提高企业退休职工和城乡居民基础养老金、优抚对象抚恤和生活补助、城乡低保对象补助水平。进一步规范城乡低保管理，全面建立城乡居民临时救助和重特大疾病救助制度，实现低保与社会救助制度的有效衔接。高度重视养老问题，重点做好以居家为基础、社区为依托、机构为支撑的社会养老服务体系建设。继续做好7类“老字号”群体、上岸定居渔民生活保障工作。完善最低工资标准调整机制，结合机关事业单位养老保险制度改革，提高基本工资标准并建立正常调整和增长机制。
　　**加大扶贫攻坚力度。**认真实施《大别山片区区域发展与扶贫攻坚规划（2011-2020年）》，减少贫困人口14万人。创新扶贫工作机制，着力推进精准扶贫。继续实施“单位包村、干部包户”定点帮扶制度，强化财政扶贫资金监督管理。加强贫困地区基础设施建设，进一步改善群众生产生活条件。加大产业扶贫力度，支持贫困县区培育优势产业。全面实施教育扶贫工程，加大贫困人口转移就业培训，增强群众致富能力。继续推进金寨“5+1”项目，充分发挥其示范引领作用。
　　（八）构建和谐社会
　　**协调发展社会事业。**着力培育和践行社会主义核心价值观，大力开展群众性精神文明创建活动。完善公共文化基础设施，加快建设市“两馆一院”，健全县“两馆一场”，推进综合性文化服务中心、农民文化乐园、社区文化活动室等建设。大力实施“四个一”品牌工程，推出一批更多体现六安特色的文艺作品。加强文化遗产保护、传承和利用，大力发展新闻广电出版事业。认真实施农村薄弱学校改造工程和第二期学前教育三年行动计划；继续推进义务教育阶段学校标准化建设，完成义务教育均衡县（区）年度验收任务；推进教育信息化。充分发挥市级统筹和大别山职教集团引领作用，积极推进中高职衔接，加快发展现代职业教育。实施县域医疗服务能力提升工程三年行动计划，促进社区卫生服务模式转变，强化妇幼保健体系建设，切实加强重大传染病防控，深入开展爱国卫生运动。广泛开展全民健身运动，开工建设市体育场馆。加强出生人口性别比综合治理，继续稳定适度低生育水平。扎实做好统计、物价、气象、人防、地震、对台、地方志、档案、残联、外事侨务、妇女儿童等工作。
　　**提升社会治理水平。**加大普法宣传力度，引导全民自觉守法、遇事找法、解决问题靠法。深入开展矛盾纠纷排查调处工作，推进人民调解、行政调解、司法调解衔接联动。建立网上受理信访制度，健全就地依法解决群众合理诉求机制。加强流动人口和特殊人群服务管理，切实做好社区矫正和安置帮教工作。全面贯彻党的民族宗教政策，依法加强宗教事务管理，促进民族团结、宗教和谐。打造平安六安，加快建设城区报警与视频监控系统，启动社会服务管理信息化工程；依法严密防范和惩治各类违法犯罪活动，确保社会安定有序、人民安居乐业。
　　**加强公共安全体系建设。**健全防灾减灾救灾体制机制，加强应急专业队伍和装备建设，提高突发事件处理能力。推进市场监管体系建设，严厉打击各种扰乱市场经济秩序的不正当行为，维护公平有序的市场环境。严格食品药品安全监管，强化检验检测能力建设，保障人民群众食品药品安全。健全完善安全生产责任体系，严格落实党政同责、一岗双责、齐抓共管要求，突出企业主体责任，切实抓好重点行业和领域的安全生产监管，坚决遏制重特大安全事故发生。加强国防动员、民兵预备役工作，深入开展双拥共建，积极争创“全国双拥模范城”，促进军民融合深度发展。
　　三、全面加强法治政府建设
　　适应经济发展新常态，实现六安发展新跨越，对政府工作提出了新的更高要求。我们要按照党的十八届四中全会的决策部署，忠实履行宪法和法律赋予的神圣职责，坚持在党的领导下，在法治轨道上开展工作，加快建设职能科学、权责法定、执法严明、公正公开、廉洁高效、守法诚信的法治政府。
　　**强化法治意识，提高履职能力。**大力弘扬社会主义法治精神，完善国家工作人员学法用法制度，引导政府系统工作人员牢固树立法治意识，时刻牢记法律红线不可逾越、法律底线不可触碰，提高运用法律手段管理经济和社会事务能力。加强政府法制机构建设，积极推行政府法律顾问制度，加强行政复议工作，坚决纠正违法或不当行政行为，认真做好行政应诉。强化干部教育培训，不断提高公务员队伍素质。
　　**科学民主决策，严格规范执法。**健全政府决策机制，把公众参与、专家论证、风险评估、合法性审查、集体讨论决定作为重大行政决策法定程序。建立并严格执行行政机关内部重大决策合法性审查机制，做到依法依规、集体决策、公开透明、终身负责。依法接受同级人大及其常委会监督，认真执行人大决议决定，积极办理人大代表议案。主动接受政协民主监督，认真办理政协委员提案，广泛听取各民主党派、工商联和无党派人士意见。高度重视社会公众和新闻舆论监督，充分发挥工会、共青团、妇联等人民团体的重要作用。坚持规范公正文明执法，完善行政处罚自由裁量权基准制度，严格实行行政执法人员持证上岗和资格管理制度，严格执行罚缴分离和收支两条线管理制度。加强执法监督，全面落实行政执法责任制。
　　**着力改进作风，提升行政效能。**巩固和发展党的群众路线教育实践活动成果，坚决防止“四风”问题反弹，真正形成作风建设新常态。切实改进文风会风，大力精简会议文件，坚决控制各类检查评比；厉行勤俭节约，严控“三公”经费支出，完成公务用车制度改革。启动政务服务业务及电子监察三级联动项目，全面推进政务公开，进一步提高政府公信力。加大机关效能跟踪问效力度，继续推进“电视问政”、“万人评科长”、“社会评窗口”工作，大力整治“庸、懒、散”行为，切实提高行政效能。
　　**坚持廉洁从政，树立良好形象。**加强反腐倡廉制度建设，全面落实党风廉政建设责任制，严格执行廉政准则，以反腐倡廉实效取信于民。规范招投标秩序，严格公共资源交易监督管理。强化行政监察和审计监督，加强领导干部和国有企业领导人员经济责任审计，加大重点项目、重点领域专项审计，强化审计结果运用。加强廉洁自律突出问题专项监督检查力度，坚决查处违法违纪案件。注重以身边典型案例开展警示教育，保持惩治腐败的高压态势，树立清正廉洁的良好形象。
　　**各位代表，**新的征程已经开启，前进号角催人奋进。让我们在中共六安市委的坚强领导下，团结一致，齐心协力，真抓实干，锐意进取，奋力谱写绿色发展新篇章，为加快幸福六安建设而努力奋斗！
